# Multi-level resistive switching in hafnium-oxide-based devices for neuromorphic computing

**DOI:** 10.1186/s40580-023-00392-4

**Published:** 2023-09-14

**Authors:** Markus Hellenbrand, Judith MacManus-Driscoll

**Affiliations:** Department of Materials Science & Metallurgy, University of Cambridge, 27 Charles Babbage Rd, Cambridge, CB3 0FS UK

## Abstract

In the growing area of neuromorphic and in-memory computing, there are multiple reviews available. Most of them cover a broad range of topics, which naturally comes at the cost of details in specific areas. Here, we address the specific area of multi-level resistive switching in hafnium-oxide-based devices for neuromorphic applications and summarize the progress of the most recent years. While the general approach of resistive switching based on hafnium oxide thin films has been very busy over the last decade or so, the development of hafnium oxide with a continuous range of programmable states per device is still at a very early stage and demonstrations are mostly at the level of individual devices with limited data provided. On the other hand, it is positive that there are a few demonstrations of full network implementations. We summarize the general status of the field, point out open questions, and provide recommendations for future work.

## Introduction

It has been estimated that by 2030, Information and Communication Technologies (ICT) will account for up to 30–50% of global electricity consumption [1], and a large portion of this is due to various inefficiencies with respect to power consumption and speed of the memory components in these technologies [2]. The large mismatch between processing and memory speed is often called ‘memory wall’, ‘memory bottleneck’, or ‘von Neumann bottleneck’, and to tackle it, new forms of computer memory are being heavily researched. Among the most mature approaches to such new forms of memory are ferroelectric (FE), phase-change (PC), and resistive switching (RS) memory. While these three technologies can be considered as relatively ‘mature’ among emerging memory technologies, research is still very much ongoing, and their maturity is still far from established memory technologies such as Flash and SRAM/DRAM.

Every year, multiple review papers are being published on various aspects of emerging memory and computing technologies [3–19], all of which cover a wide range of ongoing developments. From these reviews, it is clear that all emerging memory technologies have their strengths and weaknesses, and no single most promising new memory technology is yet on the horizon. Because of the outstanding challenges, but also because of the continuously strong performance and evolution of established memory technologies, especially NAND Flash, which emerging memories were targeted to replace in the early stages of research, a possible market entry of emerging memories faces steep thresholds [20]. A recent example of this is Intel abandoning their XPoint ‘Optane’ PC memory series and effectively merging it into a quad-level-cell NAND product.

Since replacing conventional memory technologies requires a number of high specification targets to be met, e.g., retention of more than ten years for Flash replacement or extreme endurance performance of >10^15^ for SRAM/DRAM replacement, research efforts on emerging memory technologies are thus also focusing on the ever-faster-increasing areas of machine learning and so-called artificial intelligence based on neuromorphic computing. In these areas, depending on the specific application, some of the specifications of conventional memory, e.g., retention and endurance, are less tight, even though others are more, e.g., resistance levels need to be controllable gradually rather than simply in a binary fashion. Such gradual manipulation can critically enable in-memory computing applications, which removes the von Neumann bottleneck of having to move data between memory and processing units. Thus, the aim for such devices is mimicking synaptic-like behavior as in the brain, ideally at similarly low power levels.

In this review, we add to the aforementioned excellent overviews with a more focused summary of multi-level RS in hafnium-oxide-based devices. We focus on hafnium oxide, because it is firmly established in the CMOS industry as a transistor gate dielectric. As the CMOS industry is extremely reluctant to adopt new materials into their processes, this gives hafnium oxide an edge over many other RS materials. In addition, hafnium oxide can be used both for RS and for FE applications, which presents another possible advantage.

**Fig. 1:**
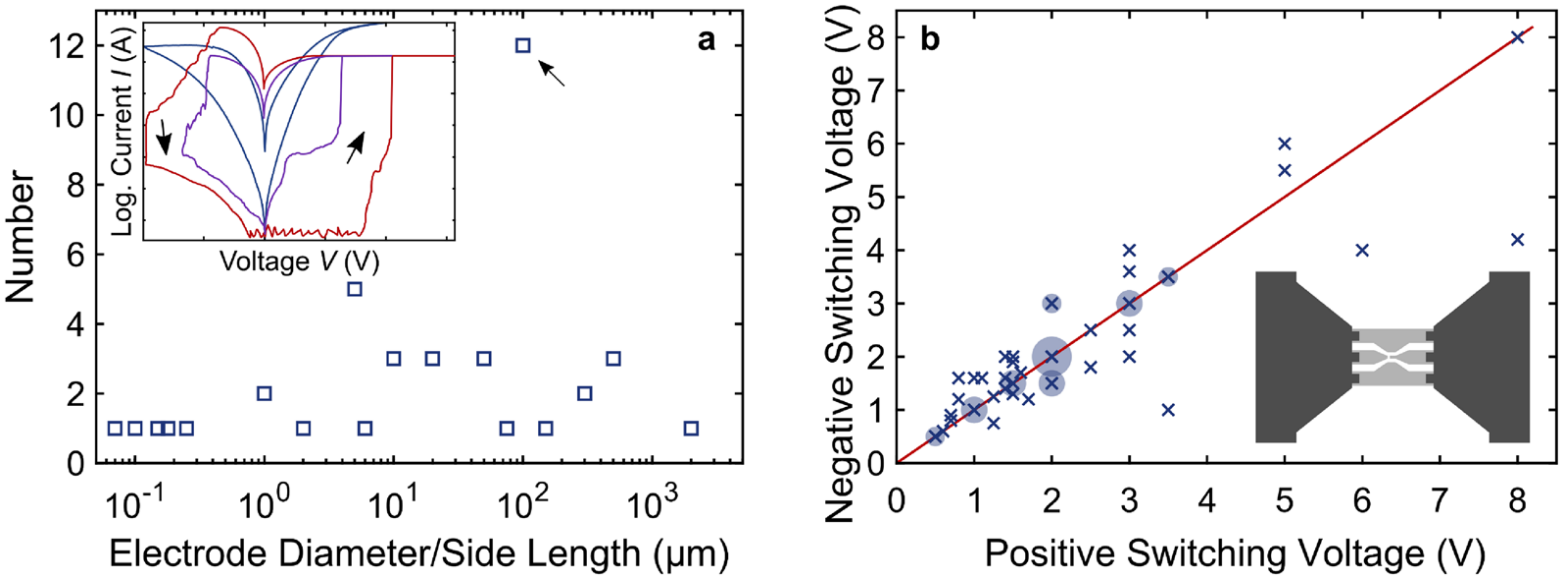
Reported device sizes and positive and negative switching voltages for hafnium-oxide-based multi-level RS. **a **Number of reported devices vs. device size. If different device sizes were reported in a publication, we included the smallest one in the figure. The arrow points out the most common device size. Inset: Illustration of gradual and abrupt switching. Red and purple, schematic: forming and subsequent switching with a positive compliance current as typically observed for filamentary RS. Blue: gradual non-filamentary switching from a system like [36]. **b** Switching voltages after forming (where necessary). Crosses correspond to individual reports, bubbles to multiple identical values from different reports, and the red line indicates symmetry between positive and negative switching voltages. The values in the figure correspond to the voltages applied to devices during the *IV* sweeping. Inset: Illustration of probes (dark gray) and probe pads (light gray) required for high-speed electrical measurements such as for measuring the ultimate device switching speed or switching power consumption

In a RS device, the application of different SET and RESET voltages to the device changes its resistance from a high resistance state (HRS) to a low resistance state (LRS), respectively, and vice versa. Examples of the resulting current–voltage (*IV*) characteristics of these processes under continuous voltage sweeps are illustrated in the inset of Fig. 1a. The different resistance states can then be used to store information.

Curiously, multi-level RS in hafnium oxide seems to be divided into two very different states. The vast majority of reports on multi-level RS in hafnium oxide are on a proof-of-concept level without significant device performance statistics. Yet there are a number of reports which already demonstrate system-level integration of multi-level devices and demonstrate complex in-memory computing tasks. Here, we discuss the materials and device aspects of gradual RS in hafnium oxide, as well as a brief excursion into advanced systems demonstrations.

We limit the review to the past 5 years so as to provide a perspective on the most recent developments of the field. The vast majority of investigations into RS in hafnium oxide have been concerned with synaptic applications, and so we focus on this aspect. But we also include a brief section on neuronal and selector devices. Finally, we give a brief overview over some immediate competitors such as multi-level RS in other binary oxides and multi-level ferroelectric switching in hafnium oxide.

## Electrical performance

The dichotomy of proof-of-concept devices and advanced systems integration brings about different focus levels in the different reports. Naturally, advanced systems demonstrations focus on the system performance without reiterating individual device characterizations. However, most recent publications are still concerned with individual devices and the underlying materials. For an appropriate reflection of the current state of ongoing work on multi-level RS, most of the following discussions are thus about devices and materials rather than systems level demonstrations.

### Standard performance metrics for emerging memory devices

While the main objective of this review is an overview over the multi-level RS performance of hafnium oxide devices, the fundamental RS performance metrics such as switching voltages, endurance, and state retention are just as important as indicators of the maturity of the technology. Based on these metrics, the vast majority of reports on gradual RS in hafnium-oxide-based devices is at a proof-of-concept level, because standard figures of merit are not reported in statistically significant detail to enable a reliable assessment of the performance, and devices are much too large to be technically relevant. An indication of the various device sizes is provided in Fig. 1a, and most devices are larger than a few micrometers. At the apparent proof-of-concept level, this is easily explained by the challenges of the simplest form of UV lithography, where the minimum obtainable feature size is typically a few micrometers. Moving forward, the demonstration of smaller devices will become increasingly important, because large-scale network implementation will be futile if the individual devices are too large.

Out of the ∼50 recent publications on multi-level RS in hafnium oxide sampled for this review, only about a third report a switching endurance of at least 10^4^ cycles, and most do so only for two states. 10^4^ is chosen here as the threshold value, because it is the approximate minimum of NAND Flash performance [21]. For offline (or ‘ex situ’) training of neural networks, where externally computed weights are programmed into a network only occasionally, this is thus a reasonably foreseeable required value. For online (or ‘in situ’) training, higher endurance numbers will be required, because the synaptic weights need to be updated much more frequently when adjusting the weights ‘live’ during local training. If future reports on RS in hafnium oxide devices, which do not explicitly report on physics/mechanism studies, do not meet at least the minimum performance of existing technology, such as Flash endurance, it is arguable whether they are of technological interest. In particular, given the state of the art, it is meaningless to label a few hundred endurance cycles as ‘excellent’, as can still be read in some reports.

Out of the one third of reports with endurance ≥10^4^, only yet another third (i.e., only about 10% total) report any details on device-to-device uniformity, which underpins an impression that the realization of multi-level RS in hafnium oxide is still in its infancy. To move this and any RS area of research forward, we thus reiterate the necessity to move away from the demonstration of single ‘hero devices’ and to report the variability of various devices and samples. As discussed elsewhere before, omission of such studies can easily lead to overoptimistic outlooks [22].

As will be discussed in Sect. 4.2.1, almost all reported multi-level RS in hafnium oxide is based on the reversible filamentary dielectric breakdown of the switching layer, which typically results in stable state retention. As a consequence, over 40% of the sampled studies reported non-volatile state retention for at least 10^4^ s without noticeable degradation, which is promising. Another ≈15% reported retention of at least 10^3^ s, but the remaining reports included only insignificant numbers below that or did not report any retention data at all. However, out of the 40% with retention ≥ 10^4^ s, less than half reported such retention for multiple resistance states and the other half only for two states, i.e., the highest and lowest resistance state. Similar to the argument that all switching cycles need be reported for reliable endurance characteristics (as opposed to, e.g., only every 1000th cycle) [22], the stable state retention of two resistance states does not prove the non-volatility of multiple intermediate states. However, inference applications based on the muti-level capabilities of emerging RS devices suffer strongly if the multiple levels are not stable, as discussed, e.g., in [23]. It should also be noted, however, that multi-level devices which are not fully non-volatile can still be of interest to reservoir computing applications, as demonstrated, e.g., in [24], where the volatility provides critical functionality. (Note, however, that the final inference layer still needs to be non-volatile for long-term usability.)

To comply with one of the main motivations for novel memory/computing devices, low power consumption, RS devices need to operate at voltages as low as possible, ideally at or below the ≈1–2 V of state-of-the-art DDR SDRAM [25]. A summary of reported applied switching voltages is provided in Fig. 1b, and about 2/3 of the reported devices fall in the window of ≤ 2 V. However, 75% of all devices in Fig. 1b still use a compliance current during their measurement, including reports which present forming-free devices. While this can be accommodated in network designs fairly easily by a selector transistor, it also precludes any selector-free array integration. It should be noted that for filamentary devices, the switching voltage is sometimes provided as the voltage where the ultra-nonlinear current change occurs. This ultra-nonlinear increase is illustrated in the inset of Fig. 1a. However, these switching voltages are subject to fluctuations, which are typically visualized by cumulative switching probabilities and these fluctuations are one of the main challenges of filamentary cycle-to-cycle variability. In Fig. 1b we summarized the maximum voltages which were applied during the *IV* sweeps of the devices as these are the voltages which guaranteed switching, and such guaranteed voltages will have to be used for reliable circuit applications. Alongside the example of an abrupt switching curve, the inset of Fig. 1a provides an example of a more gradual *IV* curve which was obtained from a device relying on interfacial rather than filamentary switching and which may alleviate the challenges of filamentary device-to-device variability [36].

While a majority of the sampled devices switch at reasonably low voltages, this does not prove low power consumption, as the power also depends on the switching speed, i.e., the integral of the switching current at the switching voltage with respect to the switching time. It is understandable that the measured power consumption is not reported often, as the fast real-time measurement of device currents requires dedicated measurement hardware and probe pad layouts, which hare sketched out in the inset of Fig. 1b [26]. However, to prove RS superiority over conventional memory and computing architecture, the switching energy will have to be reported at some point in the process of establishing a new RS-based technology, and we recommend doing so whenever possible; it is a performance metric just like endurance, retention, and uniformity. Even if on a device level, RS consumes more energy than some of the conventional memory, the ability to perform in-memory computing tasks may provide RS with an edge on a network level. For example, it was estimated that face recognition tasks performed by a RS network can be 1000 times as energy-efficient as the same task carried out by an Intel Xeon Phi processor [27], or 17 times as efficient as a dedicated ASIC [28].

In summary of this section, we reiterate the importance of being thorough in reporting standard figures of merit for emerging memory applications such as endurance and retention, and of demonstrating small (ideally ≤0.1 µm^2^ [29]) devices as well as device-to-device reproducibility [22].

### Multilevel and synaptic performance

**Fig. 2:**
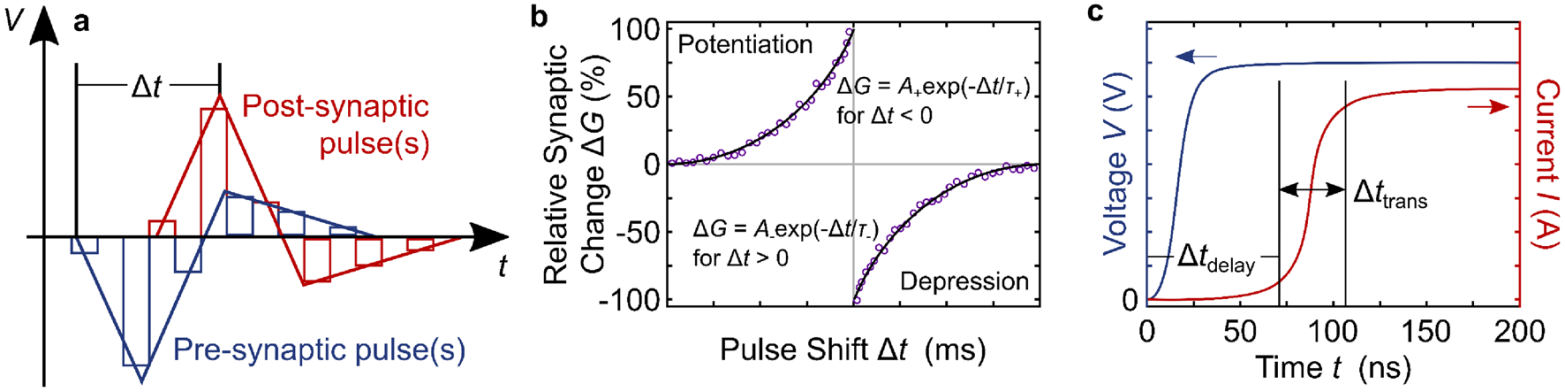
Illustration of spike-timing-dependent plasticity (STDP) and pulse timing. **a** STDP voltage profiles applied to a device. Δ*t* is shifted consecutively for each data point in **b**. In the case of a two-terminal device, one of the profiles can be inverted, added to the other, and applied on only one of the electrodes instead of the two separate electrodes. In some demonstrations, the voltage profile was applied continuously, in others as a series of pulses, in yet others only as two rectangular pulses (one positive, one negative). **b** Schematic relative change of the device conductance *G* as a function of the time shift Δ*t* in **a**. Often, Δ*G* is modelled according to the equations in the inset of **b**. **c** Timing illustration of filamentary switching as measured in [51]. After an initial delay Δ*t*_delay_ after voltage application, the current increases rapidly during Δ*t*_trans_ until it reaches the compliance value. To achieve controlled filamentary multi-level RS, the timing and amplitude of the applied voltage pulse has to be such that it stops during the rapid current transition

With regards to neuromorphic computing, arguably one of the biggest promises of RS devices over conventional memory is the ability of RS to achieve multiple, gradually tunable, often quasi-analog resistance states within a single device. The most commonly reported way of demonstrating multi-level and/or synaptic behavior in emerging RS devices is changing the resistance by a number of consecutive pulses (sometimes with consecutively increasing amplitudes), by single pulses with different amplitudes, by the compliance current, and by emulating spike-timing-dependent plasticity (STDP), where a ‘pre-’ and a ‘post-synaptic’ voltage profile are applied to different sides of a RS device with different temporal delays and the subsequent resistance change is analyzed as a function of the voltage profile overlaps with different delay times. For clarification, this is illustrated in Fig. 2a. Often, the observed response to STDP patterns follows an exponential relationship with the applied time difference Δ*t* as provided in Fig. 2b [30–35]. However, near linear dependencies have been observed, too [36].

Besides a continuous range of states, for the interpretation of the efficacy of different demonstrations, their applicability to network integrations needs to be kept in mind. This is because different from device-level demonstrations, in larger networks, the pulse patterns will have to be applied by peripheral circuitry rather than a source measure unit or parameter analyzer. With this in mind, the easiest circuit implementation to achieve synaptic behavior is by varying the numbers of pulses with constant amplitudes and pulse widths, as this can be implemented simply by gating a selector transistor and subjecting the RS device to a constant supply voltage. It is less desirable to achieve the same effect by varying pulse widths or amplitudes, as these all require additional circuit elements. Ideally, an artificial synapse should thus be programmable by a series of equal consecutive voltage pulses and the following discussion will focus on this programming scheme and its difficulties.

Specifically, unfortunately, applying a train of consecutive voltage pulses does not seem to be very effective for resistance modulation, which seems to be largely due to the complex and fast dynamics of filamentary switching, which underlies the majority (>90%) of recent reports on multi-level RS in hafnium oxide. Only three of the sampled reports demonstrated resistance variations of more than an order of magnitude when applying such a programming scheme [37–39]. In addition, only one of them was on a simple two-terminal device, whereas the others were transistors with an RS element on top or as part of their gate contact. Furthermore, for the two-terminal device, the consecutive pulses required increasing amplitudes [37], one of the transistors required high voltages of 5–10 V [38], and the other employed consecutive pulses of increasing widths [39]. In all other sampled demonstrations, the resistance modulation was less than one order of magnitude when programmed by consecutive pulses. This relative inefficacy of pulse trains is especially evident for devices which achieve ON/OFF ratios >10 in their *IV*/endurance/retention characteristics, but much lower resistance modulation when subjected to a number of identical pulses [30–33, 40–48]. While the total range of resistance modulation was not high in the sampled reports, the spacing of multiple resistance states was typically fairly tight, which implies potential for the aforementioned quasi-analog programming of these devices. Quantitatively, for almost all devices which demonstrated multi-level programming by consecutive pulses, the resistance ‘state density’ was at least 10 states per order of magnitude. (Note that in accordance with the previous discussion, this does not mean that the states covered a full order of magnitude. We opted to express a ‘state density’ because reported values appeared in vastly varying orders of magnitude and units, e.g., values were reported in units of microampere, millisiemens, kiloohm, …) Based on results from a network demonstration (more in Sect. 3), a minimum of about 10 distinguishable resistance levels can lead to close-to-baseline recognition accuracy for simple inference tasks [23], but more complex tasks may benefit from more states [28, 49].

Even though voltage pulses are not very effective for controlling resistance states over more than an order of magnitude, they can provide some insight into the switching mechanisms and dynamics of the devices. As filamentary switching is largely current- and temperature-driven [50], it makes sense that voltage pulses may not be the most appropriate means of controlling the dimensions of filaments. This is because of the complex and fast filamentary dynamics which underly the majority of RS devices (see Sect. 4.2 for further discussion) and which were resolved e.g. in [51]. In brief, for a well-controlled analog modulation of filamentary switching, the timing of the programming pulse width needs to include an initial transition delay time plus a fraction of the state transition time. If the pulse width is too short, there will only be a negligible conductance change, and if it is too long, switching will become binary. This is illustrated in Fig. 2c in accordance with the findings of [51]. The wait and transition times depend on the initial conductance state, where a higher initial conductance state leads to a shorter switching delay time, and they depend on the voltage pulse amplitude, where intuitively, a larger amplitude leads to shorter switching times. Out of the sampled publications which reported the used pulse widths, about 60% used pulse widths >1 µs. As typical filament transition times seem to be faster than that, in almost all of these reports, the used pulse amplitudes were smaller than the maximum values of the measured *IV* curves, i.e., the ‘too long’ pulses were balanced to some extent by lowering the switching voltage. This balance is nicely illustrated in several reports which plot pulsed resistance change measurements with different pulse widths and amplitudes in the same figures [31, 42, 47, 52]. In some instances of two-state endurance measurements with fast pulses (as opposed to a gradual tuning of multiple resistance states), the opposite effect could be observed, too, where the pulse amplitude had to be increased to maintain a sizeable memory window when decreasing the pulse width [53–55].

The qualitative consequence of the discussed filament dynamics can be observed in many other reports, too, where for a number of pulses to change the conductance, the first SET pulse often has a much larger effect on the conductance than any of the following pulses [31, 41–44, 47, 56–58]. And even without a disproportionately large change due to the initial pulse, RS weight update curves tend to be highly non-linear. In terms of the (normalized) conductance *G*, this non-linearity can often be described analytically as *G* = *B*_p_[1−exp(−*n*/*α*)] + *G*_min_ for potentiation, and as *G* = − *B*_d_[1−exp((*n*−*n*_max_)/*α*)] + *G*_max_ for depression, where *n* is the number of pulses, *B* and *α* are fitting parameters, where *α* indicates the degree of non-linearity and for linear weight updates, *α* = 0 [58]. Subscripts p, d, min, and max indicate potentiation, depression, minimum, and maximum values, respectively. While only a handful of the sampled reports provided a value for *α*, more than 70% of the reported weight update curves followed this exponential dependence at least qualitatively. The remaining reports achieved (near-)linear [33, 58, 59] weight updates or exhibited exponential or unclear dependencies on the pulse number.

The origin of the initially discussed discrepancy in memory window between many *IV*/endurance/retention figures and pulsed resistance modulation is thus clear. Measurements which produce a large memory window tend to cross the timing threshold of the fast filamentary switching, either by effectively applying slow consecutive pulses with increasing amplitudes during *IV* sweeps, or by employing sufficiently long and strong pulses to induce larger switching transitions. In contrast, pulsed resistance modulations often do not employ the optimum pulse shape and are consequently not able to induce similarly large conductance changes and memory windows, even after repeated pulse applications.

Qualitatively, the commonly observed non-linearity can be explained by the filament dynamics, too [58], and may in fact be a fundamental roadblock to achieving linear resistance updates in filamentary devices with equal subsequent pulses. For the SET process, the largest resistance changes occur when the filament changes from disconnected to connected, while the subsequent further strengthening of the filament causes smaller incremental changes. For the RESET process with opposite voltage polarity, the initially high current and consequentially high local temperature cause high ionic mobility which result in relatively large resistance changes. As the current, temperature, and thus ionic mobility decrease with the progressing RESET process, the resistance changes decrease, too. To achieve similar resistance changes at different filament configurations, different pulse shapes may thus be required, rather than simply equal subsequent ones, or the switching dynamics have to be controlled for more linear switching dynamics. In a promising example for this, a TaO_*x*_ “electro-thermal modulation layer” was used to slow down the abrupt filamentary dynamics, which enabled continuous linear weight updates between states separated by a factor 5–10 [58].

Often, weight update linearity is cited as an important factor for reliable neuromorphic training of networks. It may seem intuitive that programming a network is simplified if each programming pulse has the same effect on the device state. However, this seems to be more important for ex situ training, where each device has to be programmed to a specific conductivity to comply with the ex situ training outcome, or it may even be an artifact of a von-Neumann-like deterministic conceptualization. For in situ (or online) training, where a network finds its own training solution, networks are much more robust against non-linearity and faulty devices [60]. In some instances, so-called non-idealities such as non-linearity have even been shown to benefit neuromorphic network operation [61], and it has further been demonstrated that the non-linearity can be accommodated by dedicated programming schemes [62].

The few cases of devices with gradual *IV* curves (as opposed to clearly filamentary) reported much more linear conductance changes as a function of the number of programming pulses [33, 63, 64] but typically, the memory window in the *IV* curves is lower than for filamentary switching to begin with, so the dynamic range is similarly low as for many multi-level filamentary devices. The improved linearity can be explained in two ways. The first case is true interface switching, e.g., by the modulation of interfacial oxygen vacancy concentrations (more details in Sect. 4.2.2). Here, the process does not rely on the same self-accelerating current increase as in filamentary switching and may be more linear in time. The second case is the introduction of an effective series resistance into the switching stack by additional material layers. This series resistance may slow down the ultra-nonlinear switching process and improve the timing window for more accurate conductance control.

Different from the reports of controlling the conductance by a number of pulses, virtually all reports of STDP in hafnium-oxide-based devices only ever reported the normalized conductance change, which makes it difficult to compare these results. We are only aware of one recent publication which reports the absolute resistance change together with the normalized change [36]. Here, STDP led to up to three orders of magnitude resistance change.

Based on the reviewed results, the main challenge for hafnium oxide synapses is achieving a continuous range of resistances states over a large (e.g., ratio >10) memory window when programmed with subsequent pulses. The two aspects are there on their own, but rarely in combination. The key aspect is controlling the switching dynamics by careful materials design (more in Sect. 4) and/or getting the programming pulse timing right. For filamentary devices, as the pulse timing seems to depend dynamically on the state of the filament, a simple sequence of equal pulses may not be possible to use without engineering the switching dynamics to fit the programming scheme. Ideally, the route forward would thus be controlling the switching dynamics by materials engineering rather than by finding more elaborate programming schemes. Linear resistance updates are advantageous, but not strictly necessary.

## Systems demonstrations

Before taking a closer look at the materials and possible explanations for multi-level RS mechanisms in hafnium-oxide-based devices, we will present a brief summary of recent system-level demonstrations based on such devices. The impressive performance of such systems begs the question of whether fundamental demonstrations of multi-level RS on a device level are still of interest to further this research field. With a fully application-oriented perspective, the answer might be no, but from a perspective of research and understanding, there are still open questions about the functioning of the underlying devices, which will become clear in the mechanism discussions in Sect. 4.2.

**Fig. 3:**
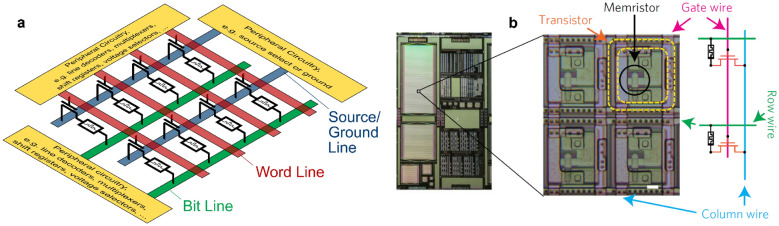
Illustration of system-level RS networks with a 1T1R configuration, which almost all systems demonstrations used. **a** Schematic to illustrate the configuration. At each intersection of one word line with the two perpendicular lines (bit line and source line), a synaptic/memory cell comprises a selector transistor and a RS element. **b** Example of an experimental demonstration, reproduced with permission from [28]. It is clear that the chip contains a considerable amount of peripheral circuitry besides the actual RS network

While there are still ongoing discussions about selector-less devices for RS networks or about which selector might be most suitable, the reality of recent system-level publications is that almost all of them used a ‘1T1R’ configuration, i.e., a selector transistor (1T) to address individual RS devices (1R) [23, 27, 28, 49, 60, 65–67]. An illustration of such a 1T1R network is provided in Fig. 3a. In the absence of a promising different approach, this may well be a hint as to how the next steps in this area will be realized. In addition, as far as can be concluded from the publications, just as for the majority of single-device demonstrations, the majority of these systems demonstrations are based on filamentary switching. In a similar line of argument as for the selector discussion, this partially rebuts the common argument that filamentary devices are not a viable way forward because of their switching non-uniformity. While the detrimental effects of a certain level of non-uniformity have been demonstrated many times (e.g., [68]), the demonstration of complex systems based on filamentary switching also proves beyond doubt that ‘it can be done’, and the results are spectacular.

The demonstrated systems comprise several 1000 devices with excellent switching uniformity and reproducibility integrated into large crossbar arrays, typically based on a commercial CMOS process in the 130 nm to 2 µm nodes. Simulations of the network sizes indicate that these large amounts of integrated devices are necessary as the recognition accuracy seems to be a strong function of the layer size [67]. In addition, the importance of the demonstration of device statistics is confirmed again by network simulations which reveal that the network accuracy is also a strong function of unresponsive devices. However, in situ training seems to be more robust to unresponsive devices than ex situ training, and networks with multiple layers more robust than single layer networks [60]. The demonstrated network architectures ranged from a single layer to 8-level 3D-integrated and fully convolutional networks including monolithic integration [67].

In most of the reports, a single thin (3.5–8 nm) layer of hafnium oxide [23, 28, 60, 65, 67, 69] or aluminum hafnium oxide [65] was used as the switching layer, as opposed to the various multi-layers and compounds in single-device demonstrations discussed in the materials overview below, and only in two reports a thicker (up to 47 nm) additional layer of tantalum oxide was used in addition to the thinner hafnium oxide or aluminum hafnium oxide [27, 49]. For the reports where the information was provided, all switching stacks were deposited by atomic layer deposition (ALD) at temperatures between 200 and 300 °C. TiN was the most commonly used electrode material [23, 27, 49, 65–67], often with a thin Ti interlayer on one side [23, 65–67], most likely to facilitate a switching redox reaction between electrode and oxide (see Sect. 4 for further discussion), and in some cases, Ta was used as one of the electrodes with Pt [60, 69] or Pd [28] as the other electrode.

Arguably, the greatest challenge to realize any systems demonstration at a university level comes after the materials optimization and even multi-layer network integration. While it is relatively straightforward to vary parameters in different deposition techniques to optimize a material, only few research groups will have the capabilities or collaborations to take the next step of integrating a materials system with the required peripheral circuitry to operate a network consisting of thousands of devices. Thus, there are only a handful of research groups who managed to demonstrate such complex systems and even among the nine examples included here, several of them involve the same groups of researchers. While this steep threshold inhibits rapid progress in the field, it makes the achieved demonstrations by just a few groups all the more impressive.

Demonstrated functionalities based on these network integrations include heart arrhythmia [23] and melanoma detection [70], the widely used recognition of handwritten digits from the MNIST database [49, 60, 65, 66, 69], facial and general image recognition, convolution, compression, and decoding [27, 28], and even video processing [69]. MNIST recognition accuracies were typically in the high 90% range close to the digital baselines and image and video processing yielded comparable (although not quantified) results as conventional software, but in real time without the processing time required for software. This includes deconvoluting compressed images [28] and edge detection in real-time videos [69]. Estimations for the improved energy efficiency of RS systems implementations ranged from 17 times as efficient as dedicated ASICs [28] to 1000 times as efficient as an Intel Xeon processor [27].

With these successful results, an important remaining question is what is limiting a wider adoption of such systems by industry. One obvious reason is that these demonstrations are fairly recent, and it takes time for such results to advance to the next level. In addition, based on the reviewed literature, two more probable reasons are the abovementioned difficulty of constructing complex peripheral circuitry and the still unresolved challenge of really good device-to-device uniformity. The latter is discussed in some of the reports on systems demonstrations themselves and is further supported by the earlier discussion of missing statistics in device level demonstrations (Sect. 2). While the summarized systems demonstrations seem to have overcome this problem for their specific materials systems, it remains a general problem of RS even after decades of research. We discuss some possible mitigations in Sect. 4.2.2.

## Materials and mechanism models

Here, we provide a summary of recent materials practices and proposed switching models. Despite recent systems demonstrations being based mostly on single-layer pure hafnium oxide or hafnium aluminum oxide, still a large number of alternative switching stack combinations are under investigation with additional materials besides hafnium oxide. Experimentally, there is little evidence for the exact switching mechanism for gradually switching hafnium oxide devices.

### Materials

About 50% of multi-level RS demonstrations based on hafnium oxide were implemented with pure hafnium oxide [30, 37, 38, 42, 44–47, 52, 54, 56, 57, 59, 71–81], another about 20% used hafnium oxide and aluminum oxide in various combinations such as bi-/multi-layers or aluminum hafnium oxide [31, 33, 47, 48, 64, 82–85], and the rest combined hafnium oxide with various other materials such as tantalum oxide [34, 55, 58, 86, 87], titanium oxide [35, 40, 51, 88, 89], tungsten oxide [53, 63, 90], molybdenum oxide [90], zirconium oxide [91], hafnium oxynitride [39], titanium oxynitride [32], or Pt nanoparticles [43], Ti [56, 92], Ni [41], or Ba [36] interspersed in hafnium oxide or aluminum oxide.

In more than 80% of the sampled reports, atomic layer deposition (ALD) or plasma-enhanced ALD was used to deposit the switching films at temperatures typically between 200 and 300 °C. In one instance, promising results were achieved by ALD at as low as 100 °C [47]. More than one third of the ALD reports did not provide the deposition temperature, which is not recommendable. Another ca. 20% of reports used (reactive/magnetron) sputtering, one report was based on e-beam evaporation [78], one on pulsed laser deposition at 400 °C [36], and one on chemical solution [77].

For about 60% of reports, at least one of the electrodes was a noble metal such as Au, Pt, or Ag, which is not ideal for CMOS compatibility, because they create deep-level traps in Si (Au), are hard to etch (Pt), or are mobile and reactive (Ag). Other than that, the most common electrode materials were Ti (ca. 50%), W (20%), and TiN (40%), where the latter was often used in combination with Ti (which is why the percentages do not add up to 100%). Amongst these, Ti stands out, as it seems to play an important role in the switching mechanism, namely facilitating the generation of oxygen vacancies in the adjacent switching layer. We discuss more on this as well as on the effects of different added layers, dopants, or particles in the following section. Already at this point, we encourage more systematic materials design studies to understand better the fundamental materials properties in the context of resistive switching.

### Proposed switching mechanisms—general discussion

Curiously, almost all (ca. 90%) demonstrations of multi-level RS in hafnia are based on filamentary switching. This is curious at first, because filamentary switching typically follows ultra-non-linear and thus very rapid and abrupt switching, intuitively at odds with the concept of gradually controlled multi-level switching. The filamentary nature of a device is often clear from the characteristic rapid current increase during the SET transition, i.e., from the high (HRS) to the low resistance (LRS) state, cf. inset Fig. 1a. Generally, in such a filamentary RS process, a highly conductive filament of oxygen vacancies is formed through the otherwise highly resistive switching layer, and the abruptness of the transition is caused by a self-accelerating effect, whereby the increasing current during initial voltage application leads to increasing local temperatures due to Joule heating, which in turn drastically accelerates the filament formation due to the exponential dependence of the oxygen vacancy mobility on the temperature [50, 93]. Often, the inherent stochasticity of filamentary RS is drawn upon as an argument for a need to find alternative mechanisms such as interfacial switching.

The systems integrations discussed before, however, demonstrate clearly that filamentary RS itself does not pose a fundamental roadblock. As only a handful of research groups have been able to demonstrate such systems, however, it seems just as clear how challenging it is to ‘get it right’. A similar argument applies to non-filamentary RS, because while there are reports on how to improve uniformity compared with filamentary RS, such improvements are clearly not strong enough either to catapult any of the candidates into a commanding lead. Thus, it is still of great importance to understand the physical details of the different switching mechanisms to improve them to a truly outstanding level.

Before taking a closer look at possible explanations of switching mechanisms, we wish to advise care about how to explain or claim the presence of a certain switching mechanism. Often, such explanations seem to hit an unfortunate middle ground between claiming strong confidence about a certain mechanism explanation, but doing so only by literature analogy (of varying detail) and without much experimental evidence or theoretical support. While it is clear that the comparison with results from the literature is a key element of scientific publications, such comparisons have to occur between appropriate results. For illustration purposes, if report X shows *IV* curves with a certain shape and relates it to a *measured* concentration change of oxygen vacancies at a certain interface, there is a logical gap if report Y observes similar *IV* curves and thus concludes the same concentration change during switching, but *without* the respective appropriate measurement to compare with. The similar *IV* curves could be caused by very different mechanisms. In short, there often needs to be more evidence for a certain mechanism besides literature comparison. This should be acquired experimentally or theoretically in the form of a quantitative model with analytic equations to back it up where possible, or ideally both by experiment and theory.

**Fig. 4:**
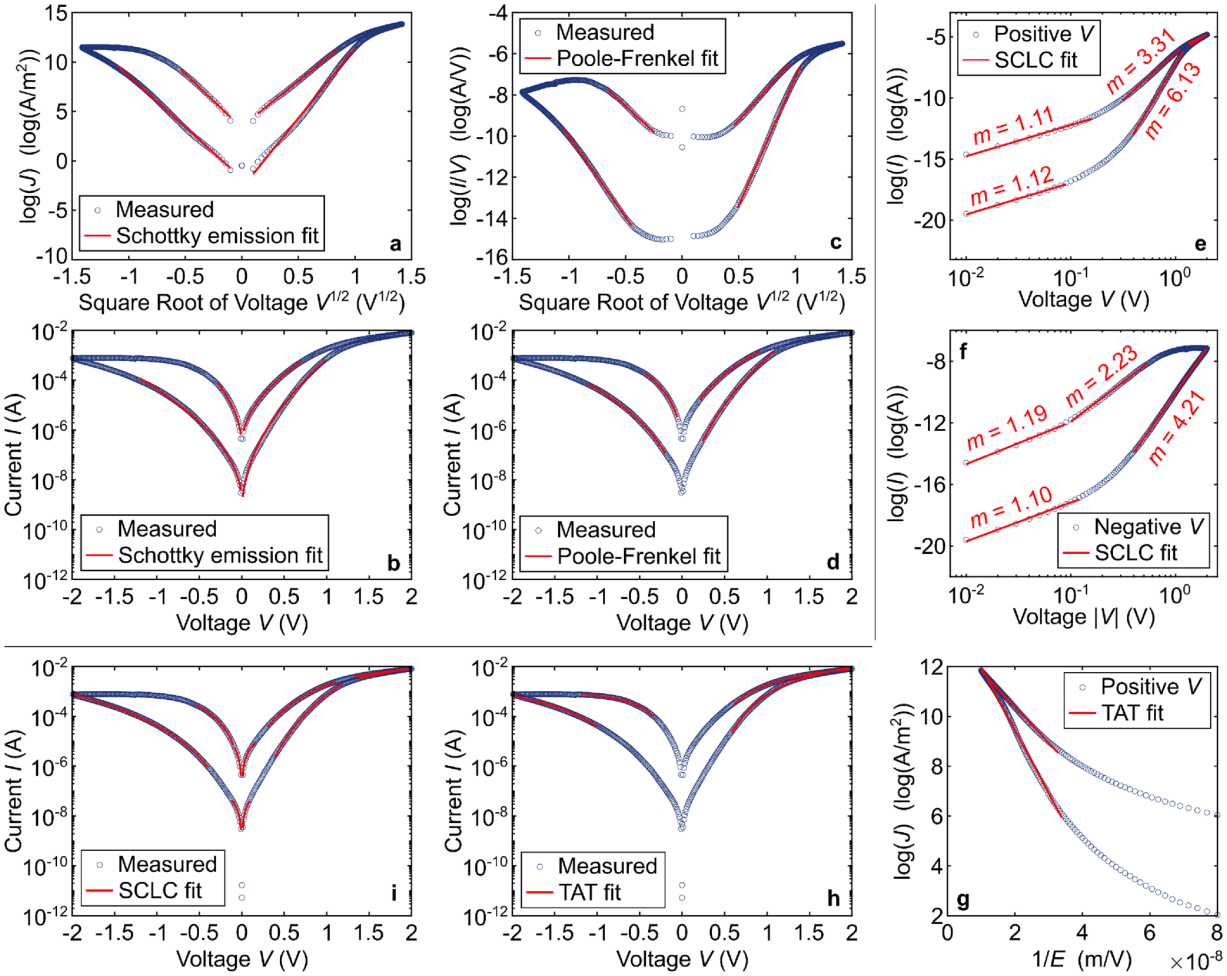
Examples of how different commonly used transport mechanisms can be fitted convincingly to the same measured *IV* curve and several regions overlap to the extent that it is impossible to make a conclusion only based on the curve fitting. The equations underlying the fits are the standard textbook equations for the respective models and the measured *IV* curve is from a system like [36]. **a**, **b** Schottky emission model, linearized fits and actual *IV* curve with overlaid fits. **c**, **d** Same for Poole–Frenkel emission. Note that for the x-axis for** c**, the negative values are −|*V*|^1/2^. The fitted areas fit just as well as with the Schottky emission model. **e**, **f** Linearized piece-wise fits for the space-charge-limited conduction (SCLC) model and **g** for the trap-assisted tunnelling (TAT) model. The TAT fits for the negative voltages are not shown due to limited figure space. **h**, **i **Measured *IV* curves with overlaid fitting results from **e**–**g**

Further on the discussion of *IV* curves, they are one of the most easily accessible starting points for mechanism investigations when supported by analytic expressions. However, we advise that the sole exercise of fitting *IV* curves with a certain transport model, which is observed regularly in the literature for hafnium oxide devices among others [33–35, 40, 53, 63, 64, 91], is not sufficient to identify unambiguously a certain switching and/or transport mechanism, despite the existence of analytic expressions. It should be self-evident that this is not sufficient especially if the *IV* characteristics are measured over several Volts, but the subsequent fitting is only carried out in a small voltage range with a few data points. An illustration of why such fitting is not sufficient is provided in Fig. 4, where different sections of the same *IV* curve could be fitted equally well by several different transport models. This is because several different analytic expressions have similar exponential dependencies on the voltage and in the case of the oft-cited space-charge-limited transport model, any region can be made to look linear in a double-logarithmic plot if only zoomed in close enough. If *IV* curve fitting is employed to understand switching/conduction mechanisms, it should be accompanied by supporting measurements, e.g., at least by temperature-dependent measurements, and/or a discussion of the physical meaningfulness of the proposed mechanism. Alternatively, any conclusion based solely on *IV* curve fitting should be kept to a general level such as the current being controlled by an energy barrier (which is true for, e.g., all four of Schottky and Poole–Frenkel emission as well as Fowler–Nordheim tunneling and trap-assisted tunneling). In addition, the fitted linearized regions as well as the original *IV* curves with overlaid calculated fits should be provided so that readers can judge for themselves how reliable the results are. Schematic diagrams of atomistic switching models without underlying modelling (e.g., density functional theory) should be avoided, too, as they easily suggest a detailed understanding of the mechanism, which may not be given.

In general, we recommend clarity about the intention of mechanism explanations. For a confident conclusion, several complementary measurements and ideally a quantitative model are required. If no such measurements or quantitative estimations are carried out, a comparison with literature results should be brief and not overclaim a certain mechanism.

#### Filamentary switching

The vast majority (ca. 90%) of sampled reports are based on filamentary switching, and the basic mechanism of reversible dielectric breakdown was summarized above. In the model of filamentary switching, different intermediate resistance states in a device can be explained by different shapes of the filaments, such as the filament diameter and/or length and the resulting resistive gap between the tip of the filament and the opposite electrode. In slightly different interpretations, the conduction path is assumed to consist of various smaller parallel filaments or dendrite-like structures [75], but resistance control by one dominant conical filament is a much more common explanation. In addition, recent findings indicate that additional weaker conduction paths in parallel with a ‘main’ filament are rather disadvantageous as they lead to noise in the current level of a targeted resistance state and thus reduce the accuracy of multi-level states [92].

It is established that oxygen vacancies play a major role in RS and in the case of filamentary switching this is forming the conductive filament. Among the sampled reports, only four relied on metal filaments through the switching layer [28, 57, 59, 94] where instead of oxygen vacancies, metal atoms form the filament.

As mentioned above, Ti and TiN were among the most commonly used electrodes and in almost 90% of these cases, the RS SET process occurred when a relative positive voltage was applied to the Ti or TiN side. Based on the relatively similar free energy of formation of hafnium oxide and titanium oxide [95], a Ti or TiN electrode or dedicated titanium oxide interlayer can act as a redox facilitator for the switching layer or as an oxygen reservoir. This is because thermodynamically, and supported by an electric field and potential temperature increase due to Joule heating, some of the oxygen from hafnium oxide in the interface region can be scavenged reversibly by Ti to form titanium oxide, leaving behind an increased concentration of oxygen vacancies and free electrons for charge compensation. This interaction between hafnium oxide and a reactive electrode or interlayer is further supported by the observation of an aluminum oxide interlayer suppressing the RS effect [33, 83].

In approaches similar to oxygen scavenging from the film by an electrode, gradients of oxygen vacancies were created in the switching layer(s) by varying conditions during deposition [45] or by depositing two oxide layers, one of which intentionally sub-stoichiometric. Examples include hafnium oxide bilayers [42, 46], or hafnium oxide combined with tantalum oxide [34] or tungsten oxide [63]. Alternatively, additional layers were introduced to act as a series resistance to slow down the self-accelerated runaway during the SET operation and allow for tuning of the filament more gradually [51, 54, 58, 64]. Hafnium oxide itself has also been used as such a series resistance with switching occurring in tantalum oxide instead [55, 86].

Also in the absence of an oxygen vacancy concentration gradient, it was demonstrated repeatedly that a controlled oxygen vacancy concentration is important to improve the RS characteristics of a film. This was demonstrated for example by the addition of Ti [56], Ni [41], or Ba [36] to the switching films, all of which increased the oxygen vacancy concentration and/or reduced their (bulk) migration activation energy. However, it should be noted that the migration barrier for oxygen inside of an oxygen-deficient filament did not change in the presence of Ti doping [56].

**Fig. 5:**
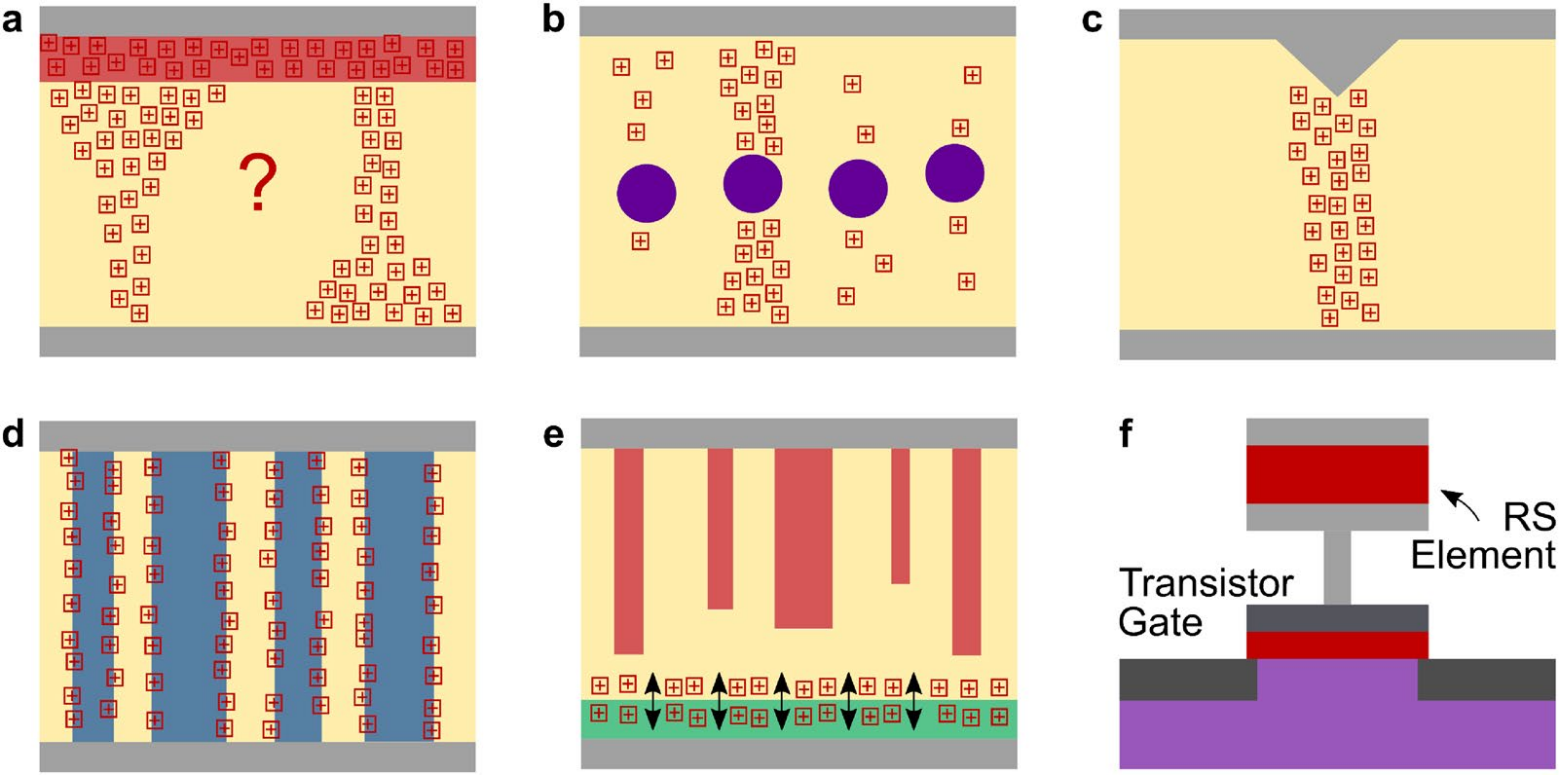
Illustrations of different proposed approaches to explain or control switching mechanisms. Such schematics should be avoided without detailed underlying understanding. Gray at the top and bottom indicate electrodes, pale yellow is the switching layer, plussed squares indicate oxygen vacancies. **a** In many publications, an oxygen-vacancy-rich layer (shaded red), either by oxygen scavenging from an electrode or a dedicated oxide layer (e.g., TiO_*x*_), is used to enhance switching properties. However, there are different explanations as to the shape of the filament and its change during filamentary switching. **b** In [43], Pt nanoparticles embedded in the switching layer were used to enhance the electric field and guide the formation of filaments. **c** In [44], nanoindentations in the top electrode enhanced the electric field underneath the indentation to guide the formation of filaments. **d** In [96], vertically aligned nanocomposite films of two different materials (HfO_*x*_ and CeO_*x*_) were used to guide filament formation along the resulting grain boundaries. **e** In [36], in a hybrid approach, vertically oriented phase separation in amorphous films was used to form effective top electrodes and restrict the switching process to the interface region (shaded green). **f** In [39], a RS element was integrated on top of a MOSFET gate contact, and the voltage division due to the RS caused large effective threshold voltage shifts and thus different source-drain currents

Besides the control of oxygen vacancy concentrations/gradients, some approaches additionally confined the formation of filaments and the movement of oxygen vacancies spatially; for example, by imbedding Pt nanoparticles inside the hafnium oxide [43] or by creating nanoindentations in the film surface [44], both to enhance the electric field, or by inducing a vertical orientation in the switching thin films to provide preferential paths for the formation of enhanced conduction channels [36, 96]. The latter was inspired by earlier complex oxide vertically aligned nanocomposite structures in which vertical interfaces acted as pre-formed electronic channels with separate ionic conduction inside the materials [97, 98]. An illustration of these approaches is provided in Fig. 5.

While the importance of oxygen vacancies seems fairly clear in most publications, open questions remain about the exact physical process of gradual RS in hafnium oxide as well as in other materials systems, and sometimes this leads to speculation or overinterpretation. For example, some explanations claim that for a conical filament, its base is closer to the reservoir side, i.e., in the case of an oxygen vacancy filament, the filament base is closer to the reactive electrode or vacancy-rich layer [33, 37, 45, 56, 88], yet other explanations claim that it is the opposite [31, 34, 40], and most of the conclusions are drawn only from electrical data.

Consequently, a similar situation presents itself with respect to explaining which aspect of the filament leads to multi-level RS in hafnium oxide. In some explanations, it is concluded that the filament gradually ruptures and recombines close to the inactive electrode [31, 37, 42, 56], in others at the interface between two different oxides in the switching layer [34, 40, 53], and in yet other cases the varying diameter of the filament is assumed to determine the multi-level resistance states [30, 33]. In some cases, it has been investigated systematically, how additional layers or dopants affect the electrical data or the switching dynamics, but often, it is also just concluded vaguely that the additional components improve the switching somehow.

It is not at all unlikely that different materials combinations result in different mechanisms or filament shapes, but this only stresses how important it is to back up detailed claims about a certain mechanism with appropriate thorough experiments or theoretical support. Among the sampled reports, examples of what such support could look like include [51, 56, 80, 99]. In particular, we encourage more systematic and detailed materials design studies to understand better the switching mechanisms and their effects on the electrical device performance.

#### Alternatives to filamentary switching—interfacial, hybrid, and transistors

Different from filamentary RS, where the switching and conduction processes are confined to the nanometer-sized filament, in interfacial switching, the resistance states are controlled by some form of interface energy barrier and electronic conduction occurs through the ‘bulk’ of the oxide. As such, the hallmark of interfacial switching is the dependence of current levels on the size of the device electrodes. In addition, the *IV* curves lack the characteristic sharp current increase due to the filament formation. In recent years, there have been scarcely few demonstrations of interfacial RS based on hafnium oxide which actually demonstrate area (or diameter) scaling [36, 63], although there are several reports which observe gradual *IV* curves [30, 33, 34, 47, 53, 54, 76, 91]. As discussed above, most commonly it is still filamentary switching at their foundation, but limited by a series resistance, even if sometimes they are called interfacial, though without providing area-dependent data. In the few presented interfacial switching devices, the mechanism is concluded to be a result of an interfacial Schottky-like energy barrier which can be tuned by the variable concentration of oxygen (vacancies) in its vicinity. Driven by the applied electric field, when oxygen vacancies accumulate near the interface, they lower the barrier height, which leads to the low resistance state. Conversely, if oxygen vacancies are depleted from the interface by the electric field, the barrier height increases to restore the high resistance state. A straightforward model explanation for this is provided, e.g., in [77].

Similar to many of the explanations for filamentary switching, a direct observation of this mechanism in hafnium oxide is missing. Also, as reported in other interfacial switching materials systems, the two demonstrations [36] and [63] suffer from poor state retention, so clearly, improvements are required to make them suitable for long-term inference applications. Alternatively, as mentioned before, the volatility could be used for reservoir computing applications [24].

Besides purely interfacial or filamentary switching, a hybrid approach was proposed recently to use only partial filaments as effective electrodes and to restrict the switching process to the interface [36]. In this approach, high levels of doping were used to control the oxygen vacancy concentration in hafnium oxide and to cause self-assembled phase separation in amorphous thin films. This phase separation led to the formation of vertically aligned nanopillars with very narrow (nanometer) lateral spacing through the upper part of the film thickness. Notably, this was achieved in amorphous films at the CMOS-friendly deposition temperature of 400 °C. The nanopillars acted as preferred paths for enhanced conduction, but because they did not reach all the way through the film thickness, they did not cause a full filamentary dielectric breakdown. Instead, they acted as the effective top electrode and restricted the switching to the bottom interface of the device stack. The combination of guided filaments with narrow spacing and interface confinement led to improved device-to-device uniformity and nanosecond switching times. An illustration of this hybrid system together with the aforementioned approaches to confine the filament formation is provided in Fig. 5.

Another alternative approach was to integrate a RS element with or on top of the gate of a metal–oxide–semiconductor field-effect transistor (MOSFET). In two cases, the explanation for RS of the source-drain current was oxygen vacancies being pushed into and out of the MOSFET channel by the electric field applied over the gate dielectric stack [38, 90]. As before, however, a direct observation of this mechanism seems to be missing. In one case, with a 65-nm-thick HfO_2-*x*_ gate dielectric and an indium zinc oxide channel, this enabled source-drain current modulations of several orders of magnitude even by subsequent pulses [38], which otherwise showed poor efficacy in two-terminal devices as discussed before. However, due to the thick gate dielectric, this required relatively large voltages of up to -10 V. In the other case of a MoO_*y*_/HfO_2_ gate stack with a WO_3-*x*_ channel, this approach resulted in very poor source-drain current modulation by subsequent pulse numbers and either, it required pulse widths of 1 s, or the modulation was less than 10% with 10 ms pulses [90].

By far the most promising transistor RS performance was demonstrated by integrating a filamentary switching element on top of the gate stack through a via contact [39]. In this approach, filamentary switching in the additional gate element was the explanation for stable multi-level retention (>10^6^ s measured without degradation) with good endurance (10^5^ measured without degradation) and almost six orders of magnitude gradual source-drain current modulation by subsequent pulses. The only potential drawback was that the pulse schemes employed increasing widths (10-70 ns) or amplitudes (up to 4.5 V). A schematic of this approach is provided in Fig. 5f.

In general, these alternative approaches face the same challenges as the filamentary demonstrations of multi-level RS in hafnium oxide. To pose as viable competitors, they will have to provide more performance statistics, smaller devices, and a better fundamental understanding of the underlying switching physics.

## Neurons and selectors

**Fig. 6:**
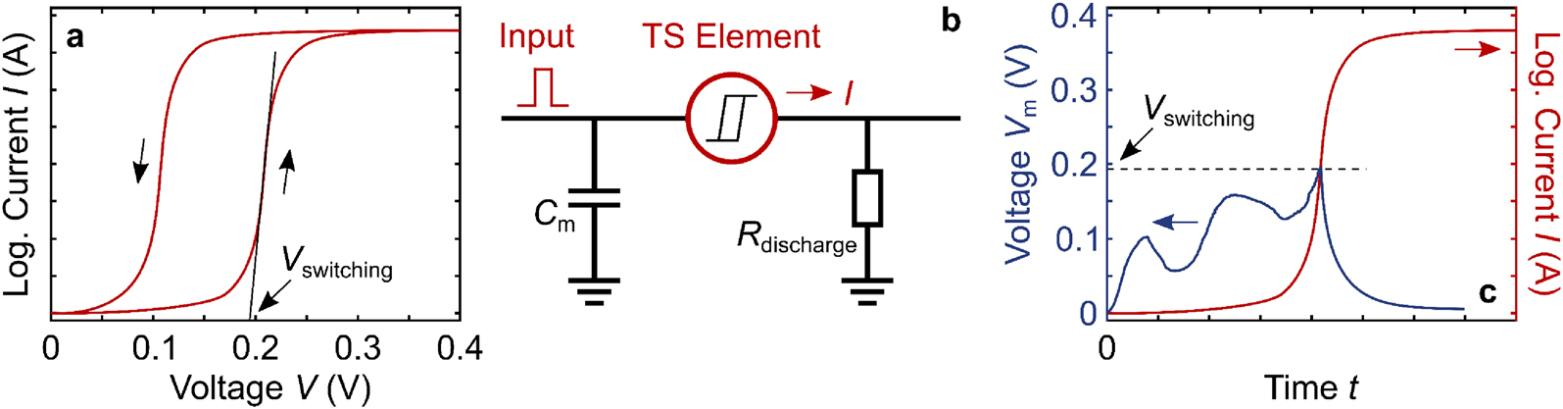
Illustration of threshold switching (TS) and neuronal functionality. **a** TS is unipolar and volatile. At a certain voltage, the current increases rapidly from the HRS to the LRS, but when the voltage is reduced, the current and the conductivity decrease back to the HRS. This could be used to implement a selector functionality to suppress sneak paths in crossbar arrays. **b** It could also be used to implement neuronal (as opposed to synaptic) functionality in this simple circuit for an integrate-and-fire neuron based on a TS device. Subsequent input pulses charge the ‘membrane’ capacitor *C*_m_. When its potential reaches the threshold *V*_switching_ of the TS element, the signal is transmitted and *C*_m_ discharges through the LRS TS element and *R*_discharge_. **c** Schematic membrane voltage *V*_m_ on *C*_m_ for an arbitrary input signal. As long as the potential on *C*_m_ remains below the switching threshold *V*_switching_, the TS device will remain insulating. Only when the switching threshold is surpassed, will a signal, such as a current to emulate neuronal firing, be possible

While in hafnium oxide, multi-level RS is mostly being investigated for (non-volatile) synaptic behavior, the material also exhibits the flexibility to enable volatile threshold switching (TS), which is highly promising for a device-(rather than circuit-)level implementation of neuronal functionality, and it holds promise for selector devices to suppress parasitic sneak paths in crossbar arrays. Different from the bipolar RS curves illustrated in the inset of Fig. 1a, TS follows a unipolar pattern and not only the current, but also the device conductivity returns to a very low level together with the voltage. This is illustrated in Fig. 6a. For neurons, TS is promising to realize an integrate-and-fire model, whereby a neuron device only ‘fires’ (sends out or transmits a signal spike) if a certain threshold is overcome [100]. This is illustrated in Fig. 6b, c, where Fig. 6b illustrates a simple integrate-and-fire neuron circuit, and Fig. 6c illustrates schematically the membrane potential *V*_m_ on the membrane capacitor *C*_m_ and the current transmitted by the TS device. For an arbitrary voltage input signal, once *V*_m_ exceeds *V*_switching_, the TS element will become conductive. This transmits a current output and at the same time discharges *C*_m_ to reset the TS element to a resistive state.

While there have been fewer demonstrations of reliable TS than of non-volatile multi-level RS, the results are very encouraging. The most promising mechanism for TS seems to be the percolation of Ag from one of the electrodes through the hafnium oxide layer under a compliance current ≤100 µA, which leads to the formation of a conductive filament to switch to the LRS, but which dissolves spontaneously when the voltage is removed. As suggested by DFT calculations, the dissolution happens because the compliance-current-limited filament is not strong enough to be thermodynamically stable. Based on DFT calculations as well, the initial Ag percolation occurs favorably along paths of oxygen vacancies. In a demonstration where devices could be switched between non-volatile RS and TS [59], a measured TS endurance of 10^4^ cycles with a memory window >10^2^ at a low switching voltage of about 0.15 V was achieved. In a similar materials system [101], >10^6^ TS endurance cycles were demonstrated with a memory window of 10^3^ at a switching voltage of about 0.2 V. Based on DFT results, this was achieved by nitrogen doping in the hafnium oxide stabilizing the formation of Ag pathways along oxygen vacancy sites. Similarly, the importance of the Ag-oxygen-vacancy interactions was concluded in [102], where a TS endurance of 10^8^ cycles was demonstrated for Ag as the active electrode with a switching voltage of 0.3 V, and an endurance of >10^9^ for an AgTe electrode with a switching voltage of 0.7 V and a memory window of ≈10^4^.

## The ‘competition’

While hafnium oxide has the advantage over other RS materials that it is already established in CMOS industry, its performance still has to stand up to competition from other CMOS-compatible materials. Thus, in the final sections of the paper, we provide a brief overview over RS in other binary oxides as well as devices based on the omnipresent ferroelectric hafnium oxide.

### Gradual RS in other binary oxides

RS in general, but also gradual and multi-level RS, is commonly observed in many other materials systems besides hafnium oxide. Besides binary oxides, this also includes, e.g., perovskites and 2D materials, but to stay within the scope of this review, we will only provide a brief summary of multi-level RS in binary oxides. The binary composition itself can be a potential benefit over other more complex materials. Some of these oxides already appeared in the discussion above when they were combined with hafnium oxide, but gradual RS has also been observed in standalone aluminum oxide [103], tantalum oxide [104], titanium oxide [105, 106], tungsten oxide [107], and various combinations thereof [108–110]. Further recent examples include silicon oxide [111], manganese oxide [112], cobalt oxide [113, 114], zinc oxide [115, 116], yttrium oxide [117], and nickel oxide [118]. Just as for the hafnium oxide devices, we limited the overview to the past few years.

The landscape of demonstrations for gradual RS in these materials presents itself almost identical to the one of hafnium-oxide-based devices, i.e., mostly at a proof-of-concept level and very few systems-level demonstrations [119]. This means mostly demonstrations of a few individual large (often ≥100 µm diameter) devices with limited statistics on performance and variation. The reported switching voltages were between ±2 and ±10 V, retention times between 10^3^ and 10^4^ s for two to four states, and the measured endurance typically ranged from 100 to about 10^4^ cycles. One notable outlier is the demonstration of 10^12^ switching cycles (switching voltage +3.5 and -7 V, two states) in a tantalum oxide/aluminum oxide device, which also exhibited multi-level RS. While >10^10^ switching cycles have been demonstrated in hafnium oxide devices, they did not show multi-level performance at the same time [120]. Just as for the discussed hafnium oxide devices, most demonstrations of neuromorphic plasticity in other oxides were based on a consecutive number of pulses, often together with paired pulse facilitation or STDP, and just as for hafnium oxide devices, the numbers of consecutive pulses did not result in strong conductance modulations, ranging from as low as 2% [118] up to a maximum factor of about 20 [108], and the (non-)linearity was comparable with hafnium oxide devices, too. Finally, explanations of switching and conduction mechanisms were on a similar level as for hafnium oxide, too, sometimes with varying degrees of speculation and only occasionally supported by substantial quantitative modelling [115, 117].

### Ferroelectric hafnium oxide

Finally, while based on a different physical mechanism, no review of RS and/or hafnium oxide these days seems to be able to go without a look at ferroelectric switching (FES) in hafnium oxide as one of the main alternatives to multi-level RS. With several review papers specifically on FES in hafnium oxide being published every year, we will only point out a few of the most recent ones [121–127] and based on these, provide a brief summary of the multi-level switching phenomena based on FES. The most common realization of FE in hafnium-oxide-based films is a polycrystalline orthorhombic thin film of hafnium zirconium oxide (HZO) formed by rapid thermal annealing [128]. However, FE has also been reported in epitaxial FE hafnium oxide in the rhombohedral phase [129, 130]. Different dopants or composites are being used to stabilize the FE phases, but HZO is by far the most commonly investigated and mature materials system.

Very generally, on the materials level, multi-level FES in hafnium oxide can be attributed to different switching dynamics of so-called domains, limited areas within a material. Such FE domains can occur due to crystalline domains, but FE domains can also stretch across crystalline domains, and there can be multiple FE domains in a monocrystalline film, and domains can be as small as half a unit cell [131]. If different domains have different coercive fields, or they can be switched sub-coercively, it is evident how a FE film can exist in different states of different FE configurations. Based on these configurations, FE can be utilized in a device in different ways.

**Fig. 7:**
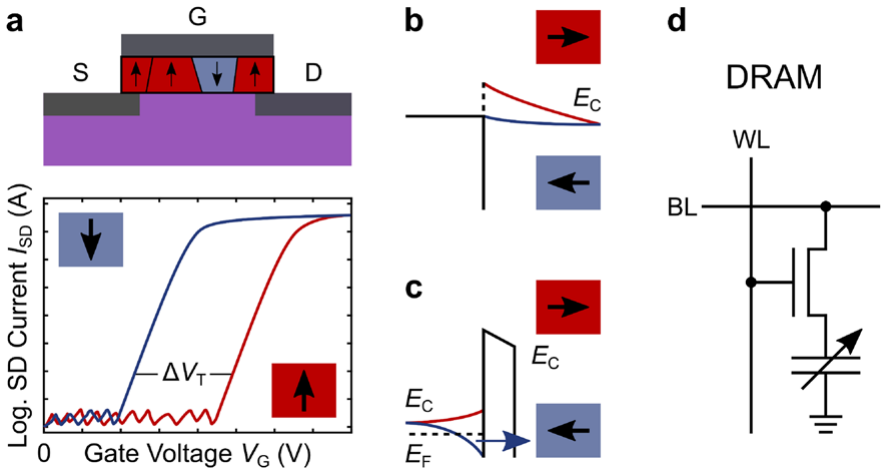
Illustrations of implementing ferroelectric switching. **a** Top: Schematic of a FE transistor (FeFET) with different domain orientations (arrows in boxes) in the gate oxide. S—source, G—gate, D—drain. Ferroelectric switching in the gate oxide will cause a shift of the transistor threshold voltage *V*_T_, creating a memory window in the transistor transfer characteristics (bottom). Whether in a three- or two-terminal device, gradual switching of different domains in a FE film can lead to multi-level states if exploited accordingly. **b** Schematic band diagrams of Schottky-to-ohmic transition device. Depending on the FE orientation, illustrated by the arrows in boxes, the contact between the FE oxide and an adjacent electrode (with an appropriate work function) can be a Schottky contact (red, increased barrier) or an ohmic contact (blue, reduced barrier), leading to a high and a low resistance state, respectively. **c** Schematic band diagrams of a FE tunnel junction. Depending on the FE orientation, electronic carriers can be accumulated (blue, band bending downwards) or depleted (red, band bending upwards) at the interface, leading to a lower or a higher resistance state due to increased/decreased tunnelling through the FE oxide from accumulated/depleted carriers at the interface. *E*_C_ conduction band energy, *E*_F_ Fermi level energy. Note that the effect of FES on the insulator was disregarded in this schematic. **d** Circuit diagram of a DRAM configuration, where the standard dielectric capacitor was replaced by a variable FE capacitor. WL—word line, BL—bit line

The most common implementation of FE hafnium oxide in devices is the FE field-effect transistor (FeFET). In this architecture, the ordinary amorphous transistor gate dielectric is replaced (typically) by HZO, and the FES induces a shift of the transistor threshold voltage, thus creating a memory window with multiple possible states based on the multi-level FES of the oxide [132]. Alternatively, FES in gate oxides can be utilized to achieve a negative capacitance in the gate stack, which can enable a sub-thermionic off-state of the transistor [133]. Possible two-terminal exploitations of FES can make use of FE tunnel junctions (FTJ) [134], Schottky-to-ohmic transitions (SOT) [135], or simply replace the dielectric of the DRAM architecture with a FE oxide. In an FTJ, the FE polarization depletes or accumulates charge carriers in a semiconductor adjacent to the FE oxide layer, leading to different conductivities across the stack. In a SOT device, the switching FE polarizations changes the Schottky barrier height between the FE oxide and the adjacent electrode and the barrier height changes the current. In a FE DRAM, the FE polarization replaces the dielectric charge as the information storage medium and due to the FE non-volatility drastically reduces the need for memory refresh events. Schematics of the different configurations are provided in Fig. 7 for illustrative clarification.

In HZO FeFETs, cycling endurances of 10^11^ and extrapolated state retention of 10 years (2 × 10^4^ s measured) have been reported for a memory window of 0.9 V with switching voltages of ±3.5 V and near-linear multi-level conductance modulation [136]. This makes FeFETs the most promising hafnium-oxide-based multi-level devices to date. For two-terminal FE capacitors, which could be used for FTJs or FE DRAM, endurances of 10^11^ have been reported, too, for switching voltages of ±3.5 V and a remanent polarization of 2*P*_r_ ≈ 30–40 µC/cm^2^ [137], but the endurance for actual FTJ demonstrations is lower than that at about 10^7^ for a memory window of ratio >10; the state retention was similarly good as in capacitors with extrapolated 10 years (10^5^ s measured) [138]. In a recently demonstrated FTJ which also exhibits multi-level synaptic functionalities, the measured endurance was approaching 10^4^ for a memory window of a factor 4 [139]. The retention was not reported in this example and the resistance update for a number of consecutive pulses followed the exponential shape discussed in Sect. 2.2.

While highly promising results have been demonstrated, they are at the same time still rare and there are still a lot of proof-of-concept demonstrations being published just as for RS devices. In the same vein, a widespread or commercial implementation of FE-based hafnium oxide devices is still missing, too, because of a number of outstanding challenges. For more comprehensive perspectives of the general field of ferroelectric hafnium oxide, we refer the interested reader to the initially mentioned dedicated reviews. The challenges most commonly identified for FE hafnium oxide are the difficulty of stabilizing a single FE phase (owing to a number of different crystal structures with similar energy), a relatively high coercive field, charge trapping related to the required applied fields, imperfect polarization screening and consequently depolarization fields which degrade state retention, as well as the wake-up and fatigue effects, which are the increase and later decrease of polarization with the number of switching cycles.

## Conclusions

Based on the summarized development of the past few years, multi-level resistive switching (RS) in hafnium-oxide-based devices has every opportunity to play an important role in taking neuromorphic and non-von Neumann computing to the next level. Similar performance values and similar shortcomings have been demonstrated at the device level among a range of materials exhibiting multi-level RS, but a clear lead is not evident. Typically reported performance values of multi-level RS hafnium oxide and other oxides are switching voltages ≤4 V, although with a majority between 0.5 and 2 V, endurances of about 10^4^ cycles, although 10^9^ have been reported for hafnium oxide and 10^12^ for tantalum oxide, measured retentions of 10^4^–10^6^ s, and ≥10 states per decade of resistance, but with pulsed multi-level resistance modulation below an order of magnitude difference. Most commonly, these values are reported for individual devices. To move the field forward, higher performance values are required and most importantly, they have to be reported on statistically significant numbers (≥100) of small (≤0.1 µm^2^) devices instead of individual large ones. In addition, fabrication temperatures should be limited to CMOS-friendly values of ≤400 °C or even better ≤350 °C, and CMOS-friendly electrode materials such as TiN or W should be used.

Along the same vein, a more thorough fundamental understanding of different switching mechanisms is still required, as many reports only provide a schematic explanation of switching mechanisms without proper experimental evidence. This need is still very much given because the main reasons for the limited multi-level resistance variation are the complex and fast filamentary switching dynamics, which the majority of multi-level RS devices are based on. This makes it clear that there is still a need for careful materials design to achieve better control of the switching dynamics. Promising approaches are based on including an additional layer to the switching stack to slow down the filamentary switching, on coupling an RS stack with a transistor, or to move over to non-filamentary switching, where several promising demonstrations have been put forward. These are typically based on creating (vertical) structure in RS thin films to define pre-existing pathways for ionic conduction or electric field control.

Among various multi-level RS oxides, hafnium oxide has the advantage that a number of large-scale systems integrations have been demonstrated, it is firmly established in CMOS industry, and it can be made ferroelectric (FE) for alternative and complimentary neuromorphic and memory applications. At the same time, FE transistors based on hafnium oxide may be the biggest competition for RS as they offer multi-level capabilities as well but much higher values for endurance and retention. FE hafnium oxide faces its own remaining challenges, however. For RS systems demonstrations, besides the fundamental device challenges which limit their more widespread implementation, the required peripheral circuitry poses a challenge for many traditionally materials-focused researchers and their realization will require considerable collaborations. The existing systems demonstrations, however, are strong indicators that multi-level RS for neuromorphic and AI applications based on hafnium oxide and similar oxides may be at the verge of commercialization. A number of startups based on these technologies consolidate this further.

## Data Availability

All data needed to evaluate the conclusions in this paper are either present in the paper or directly derived from the cited references.

## References

[1] Andrae A, Edler T (2015). On global electricity usage of communication technology: trends to 2030. Challenges.

[2] Aly MMS (2015). Energy-efficient abundant-data computing: the N3XT 1,000. Computer.

[3] Del Valle J, Ramírez JG, Rozenberg MJ, Schuller IK (2018). Challenges in materials and devices for resistive-switching-based neuromorphic computing. J. Appl. Phys.

[4] Ielmini D, Wong HSP (2018). In-memory computing with resistive switching devices. Nat. Electron..

[5] Wang Z (2020). Resistive switching materials for information processing. Nat. Rev.Mater..

[6] Xi Y (2021). In-memory learning with analog resistive switching memory: a review and perspective. Proc. IEEE.

[7] Shi T, Wang R, Wu Z, Sun Y, An J, Liu Q (2021). A review of resistive switching devices: performance improvement. Charact. Appl. Small Struct..

[8] Brivio S, Spiga S, Ielmini D (2022). HfO_2_-based resistive switching memory devices for neuromorphic computing. Neuromorphic Comput. Eng..

[9] Christensen DV (2022). 2022 roadmap on neuromorphic computing and engineering. Neuromorphic Comput. Eng..

[10] Schuman CD, Kulkarni SR, Parsa M, Mitchell JP, Date P, Kay B (2022). Opportunities for neuromorphic computing algorithms and applications. Nat. Comput. Sci..

[11] Chen S, Zhang T, Tappertzhofen S, Yang Y, Valov I (2023). Electrochemical memristor-based artificial neurons and synapses—fundamentals, applications, and challenges. Adv. Mater..

[12] Islam R (2019). Device and materials requirements for neuromorphic computing. J. Phys. Appl. Phys.

[13] Munjal S, Khare N (2019). Advances in resistive switching based memory devices. J. Phys. D. Appl. Phys..

[14] Roy K, Jaiswal A, Panda P (2019). Towards spike-based machine intelligence with neuromorphic computing. Nature.

[15] Upadhyay NK, Jiang H, Wang Z, Asapu S, Xia Q, Joshua Yang J (2019). Emerging memory devices for neuromorphic computing. Adv. Mater. Technol..

[16] Xia Q, Yang JJ (2019). Memristive crossbar arrays for brain-inspired computing. Nat. Mater..

[17] Zhang W (2019). Analog-Type Resistive Switching Devices for Neuromorphic Computing. Phys. Status Solidi Rapid Res. Lett..

[18] Kim MK, Park Y, Kim IJ, Lee JS (2020). Emerging materials for neuromorphic devices and systems. iScience..

[19] Marković D, Mizrahi A, Querlioz D, Grollier J (2020). Physics for neuromorphic computing. Nat. Rev. Phys..

[20] Chen Y (2020). ReRAM: History, Status, and Future. IEEE Trans. Electron. Devices.

[21] Salahuddin S, Ni K, Datta S (2018). The era of hyper-scaling in electronics. Nat. Electron..

[22] Lanza M (2019). Recommended Methods to Study Resistive Switching Devices. Adv. Electron. Mater..

[23] Esmanhotto E (2022). Experimental Demonstration of Multilevel Resistive Random Access Memory Programming for up to Two Months Stable Neural Networks Inference Accuracy. Adv. Intell. Syst..

[24] Du C, Cai F, Zidan MA, Ma W, Lee SH, Lu WD (2017). Reservoir computing using dynamic memristors for temporal information processing. Nat. Commun..

[25] Schlachter S, Drake B (2019). 1 A micron white paper introducing micron® ddr5 sdram: more than a generational update.

[26] Böttger U (2020). Picosecond multilevel resistive switching in tantalum oxide thin films. Sci. Rep..

[27] Yao P (2017). Face classification using electronic synapses. Nat. Commun..

[28] Li C (2018). Analogue signal and image processing with large memristor crossbars. Nat. Electron..

[29] Lanza M, Molas G, Naveh I (2023). The gap between academia and industry in resistive switching research. Nat. Electron..

[30] Ku B, Abbas Y, Kim S, Sokolov AS, Jeon YR, Choi C (2019). Improved resistive switching and synaptic characteristics using Ar plasma irradiation on the Ti/HfO_2_ interface. J. Alloys Compd..

[31] Qi M, Fu T, Yang H, Tao Y, Li C, Xiu X (2022). Reliable analog resistive switching behaviors achieved using memristive devices in AlO x/HfO xbilayer structure for neuromorphic systems. Semicond. Sci. Technol..

[32] Wang Q (2019). Interface-engineered reliable HfO2-based RRAM for synaptic simulation. J. Mater. Chem. C.

[33] Chuang KC (2019). Impact of the stacking order of HfO_x_ and AlO_x_ dielectric films on RRAM switching mechanisms to behave digital resistive switching and synaptic characteristics. IEEE J. Electron Devices Soc..

[34] Kim S, Abbas Y, Jeon YR, Sokolov AS, Ku B, Choi C (2018). Engineering synaptic characteristics of TaOx/HfO_2_ bi-layered resistive switching device. Nanotechnology.

[35] Jiang Y (2021). Linearity improvement of HfO_x_-based memristor with multilayer structure. Mater. Sci. Semicond. Process..

[36] Hellenbrand M (2023). Thin film design of amorphous hafnium oxide nanocomposites enabling strong interfacial resistive switching uniformity. Sci. Adv..

[37] Giovinazzo C, Sandrini J, Shahrabi E, Celik OT, Leblebici Y, Ricciardi C (2019). Analog control of retainable resistance multistates in Hfo_2_ resistive-switching random access memories (ReRAMs). ACS Appl. Electron. Mater..

[38] Beom K, Han J, Kim HM, Yoon TS (2021). Wide range modulation of synaptic weight in thin-film transistors with hafnium oxide gate insulator and indium-zinc oxide channel layer for artificial synapse application. Nanoscale.

[39] Hsieh ER, Chen KT, Chen PY, Wong SS, Chung SS (2021). A Forming-free HfO_2_-/HfON-based resistive-gate metal-oxide-semiconductor field-effect-transistor (RG-MOSFET) nonvolatile memory with 3-bit-per-cell storage capability. IEEE Trans. Electron Devices.

[40] Pal P (2022). Bending resistant multibit memristor for flexible precision inference engine application. IEEE Trans. Electron. Devices.

[41] Tan T (2018). The resistive switching characteristics of Ni-doped HfO_x_ film and its application as a synapse. J. Alloys Compd..

[42] Tan T, Du Y, Cao A, Sun Y, Zhang H, Zha G (2018). Resistive switching of the HfO : X /HfO_2_ bilayer heterostructure and its transmission characteristics as a synapse. RSC Adv..

[43] Algadi H, Mahata C, Alsuwian T, Ismail M, Kwon D, Kim S (2021). “Gradual resistive switching and synaptic properties of ITO/HfAlO/ITO device embedded with Pt nanoparticles. Mater. Lett..

[44] Chen Q (2019). Controlled construction of atomic point contact with 16 quantized conductance states in oxide resistive switching memory. ACS Appl. Electron. Mater..

[45] Li Z (2019). Coexistence of digital and analog resistive switching with low operation voltage in oxygen-gradient HfO_x_ memristors. IEEE Electron Device Lett..

[46] Liu C, Zhang CC, Cao YQ, Wu D, Wang P, Li AD (2020). Optimization of oxygen vacancy concentration in HfO_2_/HfO_x_bilayer-structured ultrathin memristors by atomic layer deposition and their biological synaptic behavior. J. Mater. Chem. C.

[47] Mahata C (2020). Resistive switching and synaptic behaviors of an HfO_2_/Al_2_O_3_ stack on ITO for neuromorphic systems. J. Alloys Compd..

[48] Mahata C, Kim S (2021). Modified resistive switching performance by increasing Al concentration in HfO_2_ on transparent indium tin oxide electrode. Ceram. Int..

[49] Yao P (2020). Fully hardware-implemented memristor convolutional neural network. Nature.

[50] Menzel S, Waters M, Marchewka A, Böttger U, Dittmann R, Waser R (2011). Origin of the ultra-nonlinear switching kinetics in oxide-based resistive switches. Adv. Funct. Mater..

[51] Cüppers F (2019). Exploiting the switching dynamics of HfO_2_-based ReRAM devices for reliable analog memristive behavior. APL Mater..

[52] Frascaroli J, Brivio S, Covi E, Spiga S (2018). Evidence of soft bound behaviour in analogue memristive devices for neuromorphic computing. Sci. Rep..

[53] Luo Q (2018). Self-rectifying and forming-free resistive-switching device for embedded memory application. IEEE Electron. Device Lett..

[54] Ryu JJ, Park BK, Chung TM, Lee YK, Kim GH (2018). Optimized method for low-energy and highly reliable multibit operation in a HfO_2_-based resistive switching device. Adv. Electron. Mater..

[55] Sakellaropoulos D, Bousoulas P, Nikas G, Arvanitis C, Bagakis E, Tsoukalas D (2020). Enhancing the synaptic properties of low-power and forming-free HfO_x_/TaO_y_/HfO_x_ resistive switching devices. Microelectron. Eng..

[56] Athena FF (2022). Impact of titanium doping and pulsing conditions on the analog temporal response of hafnium oxide based memristor synapses. J. Appl. Phys..

[57] Yang J, Ryu H, Kim S (2021). Resistive and synaptic properties modulation by electroforming polarity in CMOS-compatible Cu/HfO_2_/Si device. Chaos Solitons Fractals.

[58] Wu W (2018). A methodology to improve linearity of analog RRAM for neuromorphic computing. Dig. Tech. Papers Symp. VLSI Techno..

[59] Abbas H (2020). The coexistence of threshold and memory switching characteristics of ALD HfO_2_ memristor synaptic arrays for energy-efficient neuromorphic computing. Nanoscale.

[60] Li C (2018). Efficient and self-adaptive in-situ learning in multilayer memristor neural networks. Nat. Commun..

[61] Tian L, Wang Y, Shi L, Zhao R (2020). High robustness memristor neural state machines. ACS Appl. Electron. Mater..

[62] Gokmen T, Haensch W (2020). Algorithm for training neural networks on resistive device arrays. Front. Neurosci..

[63] Koroleva AA, Kozodaev MG, Lebedinskii YY, Markeev AM (2021). Interface engineering for enhancement of the analog properties of W/WO_3−x_/HfO_2_/Pd resistance switched structures. J. Phys. D. Appl. Phys..

[64] Kim S (2019). Neuronal dynamics in HfO_x_/AlO_y_-based homeothermic synaptic memristors with low-power and homogeneous resistive switching. Nanoscale.

[65] Milo V (2019). Multilevel HfO_2_-based RRAM devices for low-power neuromorphic networks. APL Mater..

[66] Valentian A (2019). Fully integrated spiking neural network with analog neurons and RRAM synapses. Tech. Dig. Int. Electron. Devices Meet. IEDM.

[67] Wu J, Mo F, Saraya T, Hiramoto T, Kobayashi M (2020). A monolithic 3D integration of RRAM array with oxide semiconductor FET for in-memory computing in quantized neural network AI applications. Symp. VLSI Technol. Dig. Tech. Papers.

[68] Chen A, Lin MR (2011). Variability of resistive switching memories and its impact on crossbar array performance. IEEE.

[69] Lin P (2020). Three-dimensional memristor circuits as complex neural networks. Nat. Electron..

[70] Lee HS (2021). Efficient defect identification via oxide memristive crossbar array based morphological image processing. Adv. Intell. Syst..

[71] Castán H (2018). Analysis and control of the intermediate memory states of RRAM devices by means of admittance parameters. J. Appl. Phys..

[72] Dueñas S, Castán H, Ossorio OG, García H (2019). Dynamics of set and reset processes on resistive switching memories. Microelectron. Eng..

[73] García H, Ossorio OG, Dueñas S, Castán H (2019). Controlling the intermediate conductance states in RRAM devices for synaptic applications. Microelectron. Eng..

[74] Ossorio OG (2021). Performance assessment of amorphous HfO_2_-based RRAM devices for neuromorphic applications. ECS J. Solid State Sci. Technol..

[75] Qi M, Guo C, Zeng M (2019). Oxygen vacancy kinetics mechanism of the negative forming-free process and multilevel resistance based on hafnium oxide RRAM. J. Nanomater.

[76] Ryu JJ, Jeon K, Yeo S, Lee G, Kim C, Kim GH (2019). Fully ‘Erase-free’ multi-bit operation in HfO_2_-based resistive switching device. ACS Appl. Mater. Interfaces.

[77] Tang ZX (2020). Analog-type resistive switching behavior of Au/HfO_2_/Zn_O_ memristor fabricated on flexible Mica substrate. Phys. E Low-Dimens. Syst. Nanostructures.

[78] Ambrosi E, Bricalli A, Laudato M, Ielmini D (2019). Impact of oxide and electrode materials on the switching characteristics of oxide ReRAM devices. Faraday Discuss..

[79] Baroni A (2022). Low conductance state drift characterization and mitigation in resistive switching memories (RRAM) for artificial neural networks. IEEE Trans. Device Mater. Reliab..

[80] González-Cordero G (2019). Analysis of resistive switching processes in TiN/Ti/HfO_2_/W devices to mimic electronic synapses in neuromorphic circuits. Solid. State. Electron..

[81] Kang H (2021). Two- and three-terminal HfO_2_-based multilevel resistive memories for neuromorphic analog synaptic elements. Neuromorphic Comput. Eng..

[82] Akbari M, Kim MK, Kim D, Lee JS (2017). Reproducible and reliable resistive switching behaviors of AlO_X_/HfO_X_ bilayer structures with Al electrode by atomic layer deposition. RSC Adv..

[83] Ismail M, Mahata C, Kim S (2022). Forming-free Pt/Al_2_O_3_/HfO_2_/HfAlO_x_/TiN memristor with controllable multilevel resistive switching and neuromorphic characteristics for artificial synapse. J. Alloys Compd..

[84] Khan SA, Kim S (2020). Comparison of diverse resistive switching characteristics and demonstration of transitions among them in Al-incorporated HfO_2_-based resistive switching memory for neuromorphic applications. RSC Adv..

[85] Wang Q (2022). Ultrathin HfO_2_ /Al_2_ O_3_ bilayer based reliable 1T1R RRAM electronic synapses with low power consumption for neuromorphic computing. Neuromorphic Comput. Eng..

[86] Ryu JH, Mahata C, Kim S (2021). Long-term and short-term plasticity of Ta_2_O_5_/HfO_2_ memristor for hardware neuromorphic application. J. Alloys Compd..

[87] Stecconi T (2022). Filamentary TaO_x_/HfO_2_ ReRAM devices for neural networks training with analog in-memory computing. Adv. Electron. Mater..

[88] Liu J (2018). An electronic synaptic device based on HfO_2_TiO_x_ bilayer structure memristor with self-compliance and deep-RESET characteristics. Nanotechnology.

[89] Ismail M, Chand U, Mahata C, Nebhen J, Kim S (2022). Demonstration of synaptic and resistive switching characteristics in W/TiO_2_/HfO_2_/TaN memristor crossbar array for bioinspired neuromorphic computing. J. Mater. Sci. Technol..

[90] Lee H (2022). Vertical metal-oxide electrochemical memory for high-density synaptic array based high-performance neuromorphic computing. Adv. Electron. Mater..

[91] Tang L, Maruyama H, Han T, Nino JC, Chen Y, Zhang D (2020). Resistive switching in atomic layer deposited HfO_2_/ZrO_2_ nanolayer stacks. Appl. Surf. Sci..

[92] Rao M (2023). Thousands of conductance levels in memristors integrated on CMOS. Nature.

[93] Ielmini D (2016). Resistive switching memories based on metal oxides: mechanisms, reliability and scaling. Semicond. Sci. Technol..

[94] Hsu CL, Saleem A, Singh A, Kumar D, Tseng TY (2021). Enhanced linearity in cbram synapse by post oxide deposition annealing for neuromorphic computing applications. IEEE Trans. Electron Devices.

[95] Sharia O, Tse K, Robertson J, Demkov AA (2009). Extended Frenkel pairs and band alignment at metal-oxide interfaces. Phys. Rev. Condens. Matter Mater. Phys..

[96] Dou H (2021). Electroforming-free HfO_2_:CeO_2_ vertically aligned nanocomposite memristors with anisotropic dielectric response. ACS Appl. Electron. Mater..

[97] Lee S, Zhang W, Khatkhatay F, Jia Q, Wang H, Macmanus-Driscoll JL (2015). Strain tuning and strong enhancement of ionic conductivity in SrZrO_3_–RE_2_O_3_ (RE = Sm, Eu, Gd, Dy, and Er) nanocomposite films. Adv. Funct. Mater..

[98] Cho S (2016). Self-assembled oxide films with tailored nanoscale ionic and electronic channels for controlled resistive switching. Nat. Commun..

[99] Dirkmann S, Kaiser J, Wenger C, Mussenbrock T (2018). Filament growth and resistive switching in hafnium oxide memristive devices. ACS Appl. Mater. Interfaces.

[100] Zhang X (2018). An artificial neuron based on a threshold switching memristor. IEEE Electron. Device Lett..

[101] Park JH, Kim SH, Kim SG, Heo K, Yu HY (2019). Nitrogen-induced filament confinement technique for a highly reliable hafnium-based electrochemical metallization threshold switch and its application to flexible logic circuits. ACS Appl. Mater. Interfaces.

[102] Banerjee W, Kim SH, Lee S, Lee S, Lee D, Hwang H (2021). Deep insight into steep-slope threshold switching with record selectivity (>4 × 1010) controlled by metal-ion movement through vacancy-induced-percolation path: quantum-level control of hybrid-filament. Adv. Funct. Mater..

[103] Qi Y, Shen Z, Zhao C, Zhao CZ (2020). Effect of electrode area on resistive switching behavior in translucent solution-processed AlO_x_ based memory device. J. Alloys Compd..

[104] Ismail M, Abbas H, Sokolov A, Mahata C, Choi C, Kim S (2021). Emulating synaptic plasticity and resistive switching characteristics through amorphous Ta_2_O_5_ embedded layer for neuromorphic computing. Ceram. Int..

[105] Choi SH, Park SO, Seo S, Choi S (2022). Reliable multilevel memristive neuromorphic devices based on amorphous matrix via quasi-1D filament confinement and buffer layer. Sci. Adv..

[106] Hu L, Han W, Wang H (2020). Resistive switching and synaptic learning performance of a TiO_2_ thin film based device prepared by sol-gel and spin coating techniques. Nanotechnology.

[107] Rudrapal K, Bhattacharya G, Adyam V, Roy Chaudhuri A (2022). Forming-free, self-compliance, bipolar multi-level resistive switching in WO_3–x_ based MIM device. Adv. Electron. Mater..

[108] Chen WJ, Cheng CH, Lin PE, Tseng YT, Chang TC, Chen JS (2019). Analog resistive switching and synaptic functions in WO_x_/TaO_x_Bilayer through redox-induced trap-controlled Conduction. ACS Appl. Electron. Mater..

[109] Kwon S, Kim MJ, Chung KB (2021). Multi-level characteristics of TiO_x_ transparent non-volatile resistive switching device by embedding SiO_2_ nanoparticles. Sci. Rep..

[110] Lee MJ (2018). Reliable multivalued conductance states in TaO_x_ Memristors through oxygen plasma-assisted electrode deposition with in situ-biased conductance state transmission electron microscopy analysis. ACS Appl. Mater. Interfaces.

[111] Park M, Park J, Kim S (2022). Compatible resistive switching mechanisms in Ni/SiO_x_/ITO and application to neuromorphic systems. J. Alloys Compd..

[112] Ai R, Zhang T, Guo H, Luo W, Liu X (2022). Multilevel resistive switching and synaptic behaviors in MnO-based memristor. Curr. Appl. Phys..

[113] Dongale TD, Khot AC, Takaloo AV, Son KR, Kim TG (2021). Multilevel resistive switching and synaptic plasticity of nanoparticulated cobaltite oxide memristive device. J. Mater. Sci. Technol..

[114] Iqbal S, Kumar M, Sial QA, Duy LT, Seo H (2022). Thermal nanostructuring for rectifying resistive switching behaviors of cobalt oxide neuromorphic devices. ACS Appl. Electron. Mater..

[115] Kumar M, Abbas S, Lee JH, Kim J (2019). Controllable digital resistive switching for artificial synapses and pavlovian learning algorithm. Nanoscale.

[116] Patil VL (2020). Bipolar resistive switching, synaptic plasticity and non-volatile memory effects in the solution-processed zinc oxide thin film. Mater. Sci. Semicond. Process.

[117] Petzold S (2020). Tailoring the switching dynamics in yttrium oxide-based rram devices by oxygen engineering: from digital to multi-level quantization toward analog switching. Adv. Electron. Mater..

[118] Zhang L (2022). Synaptic and resistive switching behaviors in NiO/Cu_2_O heterojunction memristor for bioinspired neuromorphic computing. Appl. Surf. Sci..

[119] Bayat FM, Prezioso M, Chakrabarti B, Nili H, Kataeva I, Strukov D (2018). Implementation of multilayer perceptron network with highly uniform passive memristive crossbar circuits. Nat. Commun..

[120] Lee HY (2010). Evidence and solution of over-RESET problem for HfO_X_ based resistive memory with sub-ns switching speed and high endurance. Tech. Dig. Int. Electron. Devices Meet. IEDM.

[121] Mikolajick T, Slesazeck S, Park MH, Schroeder U (2018). Ferroelectric hafnium oxide for ferroelectric random-access memories and ferroelectric field-effect transistors. MRS Bull..

[122] Mulaosmanovic H, Breyer ET, Dünkel S, Beyer S, Mikolajick T, Slesazeck S (2021). Ferroelectric field-effect transistors based on HfO_2_: a review. Nanotechnology.

[123] Li Z (2022). Ferroelectric hafnium oxide films for in-memory computing applications. Adv. Electron. Mater..

[124] Lederer M, Lehninger D, Ali T, Kämpfe T (2022). Review on the microstructure of ferroelectric hafnium oxides. Phys. Status Solidi Rapid Res. Lett..

[125] Noheda B, Nukala P, Acuautla M (2023). Lessons from hafnium dioxide-based ferroelectrics. Nat. Mater..

[126] Sharma P, Seidel J (2023). Neuromorphic functionality of ferroelectric domain walls. Neuromorphic Comput. Eng..

[127] Mikolajick T, Park MH, Begon-Lours L, Slesazeck S (2023). From ferroelectric material optimization to neuromorphic devices. Adv. Mater..

[128] Böscke TS, Müller J, Bräuhaus D, Schröder U, Böttger U (2011). Ferroelectricity in hafnium oxide thin films. Appl. Phys. Lett..

[129] Wei Y (2018). A rhombohedral ferroelectric phase in epitaxially strained Hf_0.5_ Zr_0.5_ O_2_ thin films. Nat. Mater..

[130] Wang Y (2023). A stable rhombohedral phase in ferroelectric Hf(Zr)_1+x_O_2_ capacitor with ultralow coercive field. Science.

[131] Lee HJ (2020). Scale-free ferroelectricity induced by flat phonon bands in HfO_2_. Science..

[132] Mulaosmanovic H (2017). Novel ferroelectric FET based synapse for neuromorphic systems. Dig. Tech. Papers Symp. VLSI Technol..

[133] Hoffmann M, Salahuddin S (2021). Ferroelectric gate oxides for negative capacitance transistors. MRS Bull.

[134] Ryu H, Wu H, Rao F, Zhu W (2019). Ferroelectric tunneling junctions based on aluminum oxide/ zirconium-doped hafnium oxide for neuromorphic computing. Sci. Rep..

[135] Müller ML, Becker MT, Strkalj N, MacManus-Driscoll JL (2022). Schottky-to-Ohmic switching in ferroelectric memristors based on semiconducting Hf0.93Y0.07O_2_thin films. Appl. Phys. Lett..

[136] Liao CY (2022). Endurance > 1011cycling of 3D GAA nanosheet ferroelectric FET with Stacked HfZrO_2_to homogenize corner field toward mitigate dead zone for high-density eNVM. Dig. Tech. Pap. Symp. VLSI Technol..

[137] Cao R (2019). Improvement of endurance in HZO-based ferroelectric capacitor using Ru electrode. IEEE Electron. Device Lett..

[138] Goh Y, Jeon S (2018). The effect of the bottom electrode on ferroelectric tunnel junctions based on CMOS-compatible HfO_2_. Nanotechnology.

[139] Li Z (2023). CMOS compatible low power consumption ferroelectric synapse for neuromorphic computing. IEEE Electron. Device Lett..

